# Hormonal alterations due to antipsychotic-related hyperprolactinemia

**DOI:** 10.1192/j.eurpsy.2022.2020

**Published:** 2022-09-01

**Authors:** A.L. Montejo, B. Buch, M.J. López, M.T. Arias, M.D. Corrales, E. Dominguez, C. Matos, B. Cortés, Y. Santana, I. Valrriberas, J. Matías, T. Prieto, J.M. Acosta

**Affiliations:** 1 University of Salamanca, Psychiatry, Salamanca, Spain; 2 IBSAL, Neurociencias, Salamanca, Spain; 3 hospital universitario, Psiquiatría, salamanca, Spain

**Keywords:** antipsychotic, schizophrénia, prolactin, iatrogenic

## Abstract

**Introduction:**

The use of antipsychotics (APS) is essential. Despite their great efficacy, some of them are associated with an increase in prolactin levels that can lead to hormonal changes needing to be identified and managed [1,2,3]. Hormonal changes use to have clinical implications including hypogonadism, infertility and sexual dysfunction

**Objectives:**

To evaluate possible hormonal alterations and some clinical implications produced by hyperprolactinemia (HPRL) derived from the use of some antipsychotic compounds.

**Methods:**

A complete fasting blood test was performed on a sample of 113 subjects (69 men and 44 women). 54% (n = 61) showed a normal prolactin level and 46% (n = 52) showed hyperprolactinaemia ( >50ng / ml). On the global sample, 39.8% (n = 45) was treated with some hyperprolactinemic drug , mostly risperidone and paliperidone.

**Results:**

Some differences were found depending on the gender of the subjects. A highly significant inverse relationship between the values of prolactin and testosterone was found in males (p=0.020, r=-0.285). In females, increased prolactin level was significantly related to decreased cortisol values.

**Conclusions:**

Antipsychotic-related Hyperprolactinaemia ( mainly risperidone and paliperidone) is related with a decrease in testosterone levels in males and with an increase in cortisol levels in females.

**Disclosure:**

No significant relationships.

